# Cerium-Containing Bioactive Glasses Promote In Vitro Lymphangiogenesis

**DOI:** 10.3390/pharmaceutics14020225

**Published:** 2022-01-19

**Authors:** Hanyu Xie, Sha Sha, Lingbo Lu, Geng Wu, Hongbing Jiang, Aldo R. Boccaccini, Kai Zheng, Rongyao Xu

**Affiliations:** 1 Department of Oral and Maxillofacial Surgery, Affiliated Hospital of Stomatology, Nanjing Medical University, Nanjing 210029, China; xhy@stu.njmu.edu.cn (H.X.); jhb@njmu.edu.cn (H.J.); 2 Jiangsu Key Laboratory of Oral Diseases, Nanjing Medical University, Nanjing 210029, China; sasa@stu.njmu.edu.cn (S.S.); lulingbo@stu.njmu.edu.cn (L.L.); x20225@live.com (G.W.); 3 Jiangsu Province Engineering Research Center of Stomatological Translational Medicine, Nanjing Medical University, Nanjing 210029, China; 4 Institute of Biomaterials, University of Erlangen-Nuremberg, Cauerstrasse 6, 91058 Erlangen, Germany; aldo.boccaccini@fau.de

**Keywords:** bioactive glasses, nanoparticles, cerium, lymphangiogenesis, wound healing

## Abstract

The lymphatic system is crucial for the regeneration of many tissues due to its fundamental role in immune cell trafficking, protein transport, and tissue homeostasis maintenance. Strategies stimulating lymphangiogenesis can provide new therapeutic approaches for tissue repair and regeneration (e.g., chronic wound healing). Here, we explored the effects of cerium-containing mesoporous bioactive glass nanoparticles (Ce-MBGNs) on lymphangiogenesis. The results showed that the extracts of Ce-MBGNs (1, 5, or 10 wt/v%) were non-cytotoxic toward lymphatic endothelial cells (LECs), while they enhanced the proliferation of LECs. Moreover, as evidenced by the scratch wound healing and Transwell migration assays, conditioned media containing the extract of Ce-MBGNs (1 wt/v%) could enhance the migration of LECs in comparison to the blank control and the media containing vascular endothelial growth factor-C (VEGF-C, 50 ng/mL). Additionally, a tube-formation assay using LECs showed that the extract of Ce-MBGNs (1 wt/v%) promoted lymphatic vascular network formation. Western blot results suggested that Ce-MBGNs could induce lymphangiogenesis probably through the HIF-1α/VEGFR-3 pathway. Our study for the first time showed the effects of Ce-MBGNs on stimulating lymphangiogenesis in vitro, highlighting the potential of Ce-MBGNs for wound healing.

## 1. Introduction

Wound healing is a complex process, which covers four overlapping stages, i.e., hemostasis, inflammation, proliferation, and remodeling [1]. Most wound injuries can heal spontaneously. However, some undesired conditions, for example, infection and inflammation, can cause the failure of wound healing or a prolonged healing time. Disruption of the four stages can cause an extended wound healing period resulting in a chronic wound [1]. Suffering from chronic wounds can remarkably reduce the living quality of patients, which has been a considerable issue for global healthcare. Healing of chronic wounds (e.g., ulcers caused by peripheral vascular disease and diabetes) remains an unsolved medical challenge. Many factors can cause chronic wounds, but the presence of the ischemic–hypoxic condition and a pro-inflammatory microenvironment are the main causes of chronic and persistent wounds [2]. Timely inflammation resolution and a pro-angiogenic microenvironment can therefore facilitate the healing of chronic wounds.

Bioactive glasses (BGs) are amorphous inorganic materials that can bond with both hard and soft tissues. BGs are also biodegradable and biocompatible, which can promote tissue regenerative activities (e.g., osteogenesis, angiogenesis) through releasing biologically active ions [3,4]. Generally, BGs can be divided into three categories, namely silicate, phosphate, and borate BGs, according to their glass network formers (SiO_2_, P_2_O_5_, and B_2_O_3_, respectively) [5]. The incorporation of active ions can enhance or extend the biological functionalities of BGs. For example, cerium (Ce) has been included in the composition of glass to endow BGs with antibacterial and antioxidant properties [6,7]. BGs have already been applied in hard tissue (e.g., bone tissue) and soft tissue (e.g., skin tissue) regeneration approaches [3]. The impressive healing effect of BGs on skin wounds, particularly on chronic wounds, is attracting increasing attention due to their clinical potential [8,9]. The wound healing effects of BGs are mainly induced due to their proangiogenic property by releasing biologically active ions (e.g., copper, boron, cerium) that can enhance blood vessel formation and relieve ischemic conditions [9]. The mechanisms of active ions enhancing angiogenesis are various. For example, copper (Cu) can promote angiogenesis by stabilizing the expression of hypoxia-inducible factor (HIF-1α) and by stimulating the production of vascular endothelial growth factor (VEGF) of bone marrow-derived stem cells [10].

The lymphatic vascular system consists of lymph capillaries, collecting vessels, lymph nodes, trunks, and ducts, which is regarded as the secondary vascular system [11,12]. The lymphatic vascular system plays a critical role in the tissue regenerative process, as it can transport extravasated fluids containing metabolic wastes, immune cells, and macromolecules (e.g., inflammatory mediators) after an injury has taken place [11,13]. The lymphatic vascular system stems from a network of vessels that is composed of tubular-aligned lymphatic endothelial cells (LECs). LECs are the main constituent cells of the lymphatic system, and they are crucial for inflammation resolution, lipid transport, immune cell trafficking, and tissue homeostasis maintenance [14,15]. Lymphangiogenesis is a complex process that is related to the proliferation, migration, and network formation of LECs [16]. Lymphatic dysfunction can result in a variety of diseases (e.g., lymphedema, myocardial infarction) [14]. Conversely, promoting lymphatic vessel formation provides a promising strategy to reverse disease progression [15].

Wound healing is highly related to the lymphatic vascular system [17]. Enhanced lymphangiogenesis (lymphatic vessel formation and expansion) has been shown to accelerate inflammation resolution [13]. Lymphangiogenesis is being considered as an effective therapeutic target beneficial for chronic wound healing [18,19,20]. For example, gene therapy approaches aiming at promoting lymphangiogenesis have been shown to significantly reduce chronic inflammation of the skin and accelerate the healing of wounds in diabetic mice [21,22]. Biomaterials have been combined with gene-, protein- or cell-based therapies to enhance lymphangiogenesis toward promoted skin wound healing [14,18,19]. In these approaches, biomaterials mainly act as scaffolds or delivery platforms to prolong the release of proteins and retain their bioreactivity or to enhance the stability of proteins, genes, and cells [19,23,24]. Particularly, vascular endothelial growth factor C (VEGF-C) plays an essential role in lymphangiogenesis because VEGF-C can promote lymphangiogenesis by binding with vascular endothelial growth factor receptor 3 (VEGFR-3) and then enhancing the proliferation and migration of LECs [24]. VEGFR-3 can also be transcriptionally activated by hypoxia-inducible factor-1α (HIF-1α), suggesting the role of the hypoxia microenvironment in lymphangiogenesis [25].

Recent studies have shown that the sustained release of VEGF-C using hydrogels could promote LEC proliferation and migration and lymphatic vascular network formation [23,24]. However, the effects of biomaterials alone on lymphangiogenesis have been scarcely investigated to date. Given the instability and vulnerability of proteins (for example, VEGF-C) in the physiological environment, biomaterials that can intrinsically promote lymphangiogenesis are particularly attractive in the treatment of chronic wounds and lymphangiogenesis-related diseases. Numerous in vitro and in vivo experimental results have evidenced the pro-angiogenic capacity of BGs [26]. However, it is still unclear if BGs can promote lymphangiogenesis toward enhanced wound healing. Previous studies have shown that BGs can stimulate cellular activities of different cell types (including endothelial cells) involved in angiogenesis toward promoted vascularization [26]. Hence, we hypothesized that BGs could enhance the viability, proliferation, and migration of LECs and consequently promote lymphangiogenesis.

In this study, we aimed to investigate the effects of a particular formulation of BGs on in vitro lymphangiogenesis. Cerium-containing mesoporous bioactive glass nanoparticles (Ce-MBGNs) were used, considering their unique morphological characteristics (nanoscale particle size, sphere-like shape, mesoporous structure) that are beneficial for applications as rigid fillers or delivery platforms for drugs and biologically active ions [27]. Nanoscale BGs possess a larger specific surface area and surface-area-to-volume ratio than their micro-sized counterparts, which are beneficial for their applications as building blocks of composites [4]. Nanoscale BGs also maintain the biological properties of bulky BGs (e.g., micro-sized powders, porous scaffolds), as these properties are mainly induced by their dissolution products (active ions). We therefore selected nanoscale BG particles in this study. In addition, the incorporation of Ce into MBGNs can endow the particles with anti-oxidant and anti-inflammatory activities that are favorable for wound healing under inflammatory conditions [6,28]. Therefore, we evaluated for the first time the influence of Ce-MBGNs on viability, proliferation, migration, and lymphatic vascular network formation of LECs. Given the intended application of Ce-MBGNs as rigid fillers in composites (direct interactions between Ce-MBGNs and cells seldom take place), we evaluated the effects of the dissolution products of Ce-MBGNs on LECs as BGs usually exert their biological effects through released active ions during dissolution. Finally, we investigated the potential cellular pathway stimulating LECs activities induced by Ce-MBGNs.

## 2. Materials and Methods

### 2.1. Synthesis and Morphology of Ce-MBGNs

Ce-MBGNs were prepared using a two-step approach combining microemulsion-assisted sol–gel synthesis and post impregnation as reported previously [6]. Briefly, binary SiO_2_-CaO MBGNs were first synthesized as described previously [6]. Ce was then incorporated into MBGNs using an impregnation method. As prepared MBGNs were soaked in cerium nitrate solution (0.2M in ethanol) for 24 h at a concentration of 10 mg/mL (30 °C) under stirring. Ce-MBGNs were then collected by centrifugation and washed before calcination at 700 °C for 2 h. Unimpregnated MBGNs were also used for further experiments. All reagents used for Ce-MBGNs preparation were purchased from Sigma-Aldrich (Darmstadt, Germany). The chemical composition of Ce-MBGNs has been confirmed to be ~86.0 SiO_2_-11.2 CaO-2.8 CeO_2_ (mol%) in our previous study [6]. Ce-MBGNs also have a large specific surface area of ~344 m^2^/g and exhibit negative surface charge in physiological fluids [6].

Morphology of Ce-MBGNs was characterized by using a field emission scanning electron microscope (FE-SEM; Auriga, Zeiss, Oberkochen, Germany). Ce-MBGNs were dropped on conductive aluminum tapes without sputter coating for FE-SEM observation. The microstructure of Ce-MBGNS was also investigated by transmission electron microscopy (TEM; Phillips CM30, Amsterdam, Netherlands). For TEM observation, Ce-MBGNs were dispersed in ethanol by ultrasonication and dropped on Cu grids.

### 2.2. Cell Culture

SVEC4-10 cell line (American Type Culture Collection, ATCC), a mouse lymphatic endothelial cell line derived from auxiliary lymph node, was used to investigate the interaction between BGs and LECs. SVEC4-10 cell line is more stable than the primary LECs and could lead to more reliable results. SVEC4-10 cell line was maintained in Dulbecco’s Modified Eagle Medium (DMEM, Gibco, Thermo Fischer Scientific, Waltham, MA, USA) containing 10% fetal bovine serum (FBS), 100-μg/mL streptomycin, and 100-U/mL penicillin. The cells were cultured in a humidified, 5% CO_2_ atmosphere at 37 °C. When the confluence reached approximately 80%, the cells were passaged and the media were exchanged every two days. According to the purpose of experiments, different types of conditioned cell culture media were used in this study. Cell culture media containing 0.5, 1, 5, or 10 wt/v% Ce-MBGNs was used to prepare the Ce-MBGN conditioned media. We selected these concentrations of Ce-MBGNs to prepare the conditioned medium according to the results published in our previous study [6] and preliminary experimental results. To prepare the conditioned media, Ce-MBGNs were sterilized by heating at 160 °C for 2 h. The sterilized Ce-MBGNs (0.5, 1, 5, and 10 wt/v%) were soaked in the complete media for 24 h. The supernatant was then collected, filtered, and used as the conditioned media. The complete culture medium containing VEGF-C (50 ng/mL) was also used as a conditioned medium. Cell culture media without VEGF-C or Ce-MBGNs was used as a control.

### 2.3. Ionic Concentration of Conditioned Culture Medium

Inductively coupled plasma optical emission spectrometry (ICP-OES; Agilent 5110, Santa Clara, CA, USA) was carried out to determine the ionic concentration of conditioned culture media. Briefly, 100 mg of sterilized MBGNs or Ce-MBGNs were soaked in 10 mL complete culture medium located in an incubator for 24 h at 37 °C. The supernatant was centrifugally collected and filtrated (0.22 μm). Then, 1 wt/v% (10 mg/mL) conditioned media were prepared for the measurement. The concentrations of released Si, Ca, and Ce ions in conditioned media were determined using ICP-OES.

### 2.4. Cytotoxicity and Cell Proliferation Assay

In vitro cytotoxicity and cell proliferation were evaluated using the Cell Counting Kit-8 (CCK-8) assay. Briefly, LECs were digested with 0.25% trypsin to prepare a cell suspension that was seeded in a 96-well culture plate (1 × 10^4^ cells/well). After culture of LECs with complete media or conditioned media (50 ng/mL VEGF-C, extracts of 0.5, 1, 5, and 10 wt/v% Ce-MBGNs and extract of 1 wt/v% of MBGNs) for 0 day, 3, 5, and 7 days, the culture media were removed and 100 μL of serum-free DMEM containing 10 μL of CCK8 reagent was added. After incubation for a further 2 h at 37 °C, the absorbance was measured (490 nm wavelength) with a microplate reader (ELx800; BioTek Instruments, Winooski, VT, USA).

### 2.5. Fluorescence Staining

For fluorescence staining, LECs were seeded on coverslips and allowed for cell adherence. After that, the culture media were replaced with the conditioned media (50 ng/mL VEGF-C, extract of 1 wt/v% Ce-MBGNs and MBGNs) and incubated for 24 h. The cells were then fixed with 4% paraformaldehyde for 30 min and then infiltrated with 1% Triton X-100 (Beyotime, Shanghai, China) for 10 min. The cells were pre-blocked with goat serum to remove non-specific staining. The coverslips were incubated with rabbit anti-Lyve1 (lymphatic vessel endothelial hyaluronan receptor) (cat#: ab218535, 1:200, Abcam, Cambridge, UK) overnight at 4 °C. After washing with PBS, the coverslips were hatched with FITC-labeled secondary IgG for 45 min at 37 °C. The cells were stained with 4′,6-diamidino-2-phenylindole (DAPI) for 90 s at room temperature. The images were then recorded under the fluorescent microscope (Leica Microsystems, Mannheim, Germany).

### 2.6. EdU (5-Ethynyl-2′-Deoxyuridine) Staining Assay

EdU staining was performed using the EdU Cell Proliferation Assay Kit (Ribo Biotechnology, Guangzhou, China) following the manufacturer’s instruction. Briefly, LECs were seeded on the coverslips and allowed for cell adherence. After that, the culture media were replaced with conditioned media (50 ng/mL VEGF-C, extract of 1 wt/v% Ce-MBGNs and MBGNs) and cultured for 24 h. After inoculation with 100 μL EdU (50 μM) for 2 h, the cells were fixed for 30 min and successively inculcated with Glycine (2 mg/mL) and 0.5% Triton X-100 for 10 min. The coverslips were hatched with Apollo reaction cocktail for 30 min at room temperature and washed with PBS (containing 0.5% Triton X-100) 3 times. DAPI was used to stain cell nuclei before observation under a fluorescent microscope (Leica Microsystems, Mannheim, Germany).

### 2.7. Scratch Wound Healing Assay

Approximately 5 × 10^5^ LECs were seeded into a 6-well culture plate containing 2 mL complete medium/well. When the confluence reached ~90%, a 10 μL pipette tip was used to generate scratch zones by streaking the culture plate perpendicularly. The cells were gently washed with phosphate-buffered saline (PBS) 3 times, and the media were replaced with the conditioned media (50 ng/mL VEGF-C, extract of 1 wt/v% Ce-MBGNs and MBGNs). The scratch zones were observed by light microscopy (Leica DM IL, Mannheim, Germany) and images were recorded after culture for 0, 12, and 24 h to monitor the migration of cells. A relatively short culture time was selected to minimize the cell proliferation effect on cell migration.

### 2.8. Transwell Migration Assay

LECs were digested with trypsin, and 200 μL serum-free DMEM containing 5 × 10^4^ cells was added to the upper chamber of Transwell (8 µm pore-size filter membranes; Corning, NY, USA) and the conditioned media (50 ng/mL VEGF-C, extract of 1 wt/v% Ce-MBGNs and MBGNs) were added to the lower chamber. After 24 h incubation, the chamber was taken out and the culture plate was washed with PBS 3 times. The migrated cells adhering to the bottom side of the Transwell membrane were fixed with 4% paraformaldehyde for 30 min, stained with crystal violet staining solution for 20 min, and washed with PBS 3 times. The migrated cells were observed under an inverted microscope (Leica Microsystems, Mannheim, Germany). Fields of view (n = 5) were randomly selected to calculate the average number of migrated cells.

### 2.9. In Vitro Lymphatic Vascular Network Formation Assay

After thawing on ice at 4 °C overnight, 60 μL Matrigel (Corning Inc., New York, NY, USA) was added to a pre-cooled 96-well plate and subsequently incubated at 37 °C for 30 min to form a gel. LECs were seeded in each Matrigel-coated well at a density of 4 × 10^4^ cells/well and incubated in conditioned media at 37 °C for 3 h. Five fields were randomly selected to observe the tube length and network structural complexity under a light microscope.

### 2.10. Western Blot

After being cultured with complete media and the conditioned media (50 ng/mL VEGF-C, extract of 1 wt/v% Ce-MBGNs and MBGNs) for 24 h, LECs were lysed with Radio Immunoprecipitation Assay (RIPA) buffer (Beyotime, Shanghai, China) containing 1% 10 mM protease inhibitor-Phenylmethanesulfonyl fluoride (PMSF, Beyotime, Shanghai, China) for 30 min on ice. The lysed cells were then centrifuged (12,000 r/min) for 10 min at 4 °C and the supernatant was extracted. The bicinchoninic acid (BCA) method was used to quantify the protein concentration. Proteins were separated by sodium dodecyl sulfate-polyacrylamide gel electrophoresis (SDS-PAGE) under constant pressure and transferred to the polyvinylidene difluoride (PVDF) membrane (Millipore, Billerica, MA, USA) with a constant current (300 mA). The PVDF membrane was placed in 5% skim milk, blocked for 3 h, and incubated with rabbit anti-Lyve1 (cat# ab218535, 1:1000, Abcam, Cambridge, UK), rabbit anti-HIF-1α (cat#14179, 1:1000, Cell Signaling Technology, Beverly, MA, USA), Rat anti-VEGF Receptor 3 (cat# ab273148, 1:1000, Abcam), or Mouse anti-β-Actin (cat#3700, 1:1000, Cell Signaling Technology) at 4 °C overnight. After washing with TBST (Tris-buffered saline with 0.1% Tween 20) 3 times, a horseradish peroxidase-conjugated secondary antibody (1:10,000) was incubated with blot for 50 min. An ECL (electrochemiluminescence) detection kit (Thermo Fisher Scientific, Waltham, MA, USA) was used to visualize the blots.

### 2.11. Statistical Analysis

The results are presented as mean values ± standard deviation (S.D.). All experiments were repeated independently at least in triplicate (N = 3) with three technical repeats (*n* = 3). The difference between the two groups was analyzed using Students’ *t* test. Analysis across multiple comparisons was performed using one-way ANOVA with Tukey’s post hoc test. *p* < 0.05 (*), *p* < 0.01 (**) and *p* < 0.001 (***) were considered statistically significant. *p* > 0.05 (#) was considered not statistically significant.

## 3. Results and Discussion

### 3.1. Ce-MBGNs Exhibit Sphere-like Shape and Mesoporous Structure

**Figure 1:**
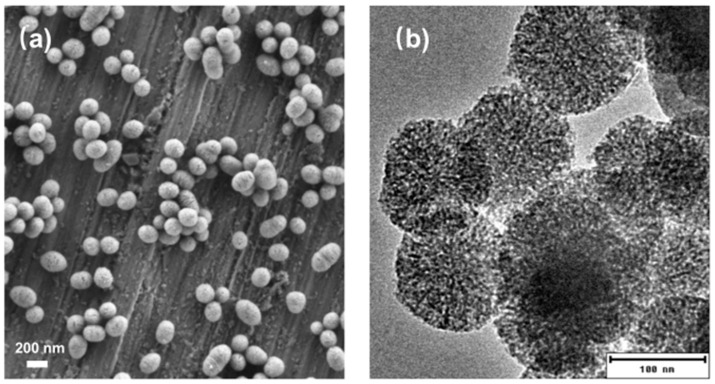
(**a**) SEM and (**b**) TEM images of Ce-MBGNs showing their spherical shape and mesoporous structure.

Figure 1 shows SEM and TEM images of Ce-MBGNs. As can be seen in Figure 1a, the nanoparticles exhibited a sphere-like shape with a particle size in the range 100–200 nm. The size and shape of the nanoparticles obtained were uniformly distributed, which is consistent with the morphology of particles synthesized using the same method [6]. However, some ellipsoidal particles could be observed in the image, which were induced by the fusion of spherical parties [29]. The presence of these ellipsoidal particles widened the size distribution of Ce-MBGNs. The TEM image (Figure 1b) shows the mesopores throughout the nanoparticles. The morphological (sphere-like shape, mesoporous structure), compositional (tunable composition), and physiological (biodegradation, antioxidant) characteristics of Ce-MBGNs make these nanoparticles promising fillers of composites, injectable gels for various therapeutic applications including wound healing. Moreover, Ce-MBGNs have antioxidant and anti-inflammatory activities that are potentially beneficial for the healing of chronic wounds caused by inflammation [30].

### 3.2. In Vitro Cytotoxicity and LECs Proliferation

**Figure 2:**
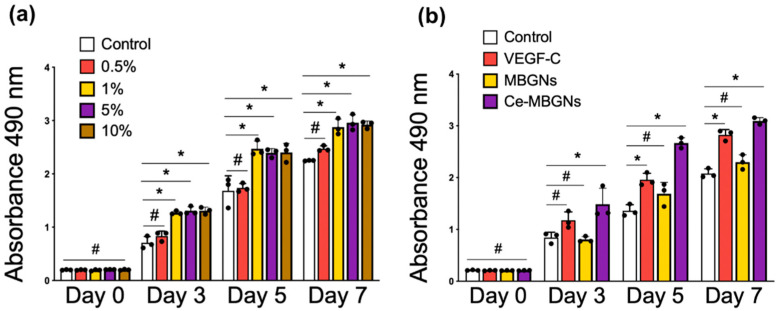
CCK-8 assay results of (**a**) LECs cultured with the conditioned media of Ce-MBGNs (0, 0.5, 1, 5, 10 wt/v% extract) and (**b**) LECs cultured with conditioned media of VEGF-C (50 ng/mL), MBGNs (1 wt/v% extract), and Ce-MBGNs (1 wt/v% extract). The experiment was performed at least in triplicate independently. Three technical repeats were used within each experiment that was repeated in triplicate. Data are presented as mean ± S.D. *p* < 0.05 (*) and *p* > 0.05 (#).

We first investigated the in vitro cytotoxicity of Ce-MBGNs toward LECs. The CCK-8 results (Figure 2a) showed that the conditioned media containing extracts of Ce-MBGNs did not reduce the viability of LECs after culture for 3 days compared to the control, as indicated by their comparable OD values, demonstrating the non-cytotoxicity of Ce-MBGNs at the tested concentrations (0.5, 1, 5, or 10 wt/v% extract). In addition, the OD values of all groups increased over time, indicating the proliferation of LECs in culture with the conditioned media of Ce-MBGNs. With the increase in culture time up to 7 days, the conditioned media at relatively high concentrations (1, 5, and 10 wt/v% extract) induced significantly higher OD values than the control, which suggested that Ce-MBGNs at suitable concentrations could enhance the proliferation of LECs. No significant difference in proliferation was observed among the different conditioned media (extract of 1, 5, and 10 wt/v% Ce-MBGNs). These results proved the non-cytotoxicity of Ce-MBGNs toward LECs. Moreover, Ce-MBGNs at concentrations of 1, 5, and 10 wt/v% could also promote the proliferation of LECs. We thus selected a 1 wt/v% concentration of particles for further experiments to find a minimal concentration of Ce-MBGNs that could still positively affect LECs responses.

VEGF-C is positively correlated with the proliferation, migration, and vascular assembly of LECs [19]. We therefore selected VEGF-C as a positive control in this study. Considering the promoting effects of VEGF-C on the viability and proliferation of LECs, we compared the effects of Ce-MBGNs (1 wt/v% extract) and VEGF-C on LECs behavior. MBGNs (Ce-free) (1 wt/v% extract) were also used as a comparison. Figure 2b shows the proliferation of LECs in culture with conditioned media containing extracts of MBGNs and Ce-MBGNs (both 1 wt/v%) as well as VEGF-C (50 ng/mL). This concentration range of VEGF-C has been shown to enhance both the proliferation and migration of LECs as well as the formation of a lymphatic vascular network [19,24]. As anticipated, under the stimulation of VEGF-C, cell proliferation (OD value) was enhanced compared to the control group and the MBGNs group. MBGNs (1 wt/v% extract) did not significantly enhance the proliferation of LECs over time, but they also had no cytotoxicity on LECs (Figure 2b). On day 5 and 7, the OD values of Ce-MBGNs group were significantly improved compared to the control and even higher than that of the VEGF-C group, indicating that the presence of Ce-MBGNs extracts (1 wt/v%) could promote LECs proliferation to a greater extent than VEGF-C (50 ng/mL).

**Figure 3:**
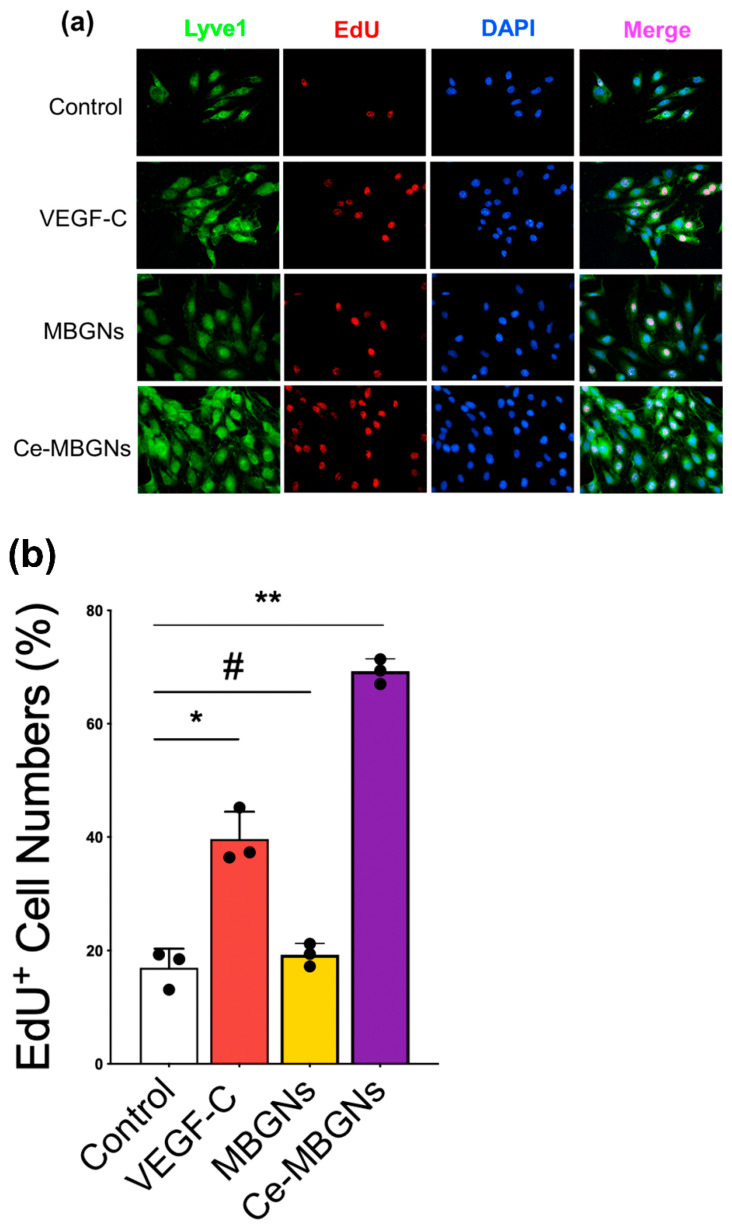
(**a**) Fluorescence images (Lyve1, EdU, DAPI) and (**b**) the quantitative EdU assay result demonstrating that the proliferation of LECs cultured in conditioned media containing Ce-MBGNs (1 wt/v% extract) was remarkedly increased compared to VEGF-C (50 ng/mL), MBGNs (1 wt/v% extract) and the control groups. Nuclei are stained with DAPI (blue). Scale bar = 50 μm. *p* < 0.05 (*), *p* < 0.01 (**) and *p* > 0.05 (#).

Figure 3a shows fluorescence images (Lyve1, EdU, DAPI) of LECs after culture with the conditioned media for 24 h. Lyve1, a cell surface receptor on LECs, was used as a cell marker to indicate LECs. As anticipated, the cells were stained by Lyve1 in all groups indicating the presence of LECs in these groups. To further evaluate the impact of Ce-MBGNs on the proliferation of LECs, the EdU assay was performed. EdU is known as a thymidine analogue that can be incorporated into the DNA of proliferated cells [31]. As seen in the representative fluorescence images, more proliferated LECs were observed in the VEGF-C and Ce-MBGNs groups in comparison to the control and Ce-free MBGN groups. More stained cells were observed in the Ce-MBGN group compared to the VEGF-C group. Figure 3b shows the quantitative analysis of EdU stained LECs. As can be seen, VEGF-C and Ce-MBGNs enhanced the proliferation of LECs; meanwhile, Ce-MBGNs could promote cell proliferation to a greater extent than VEGF-C. Taken together, the CCK8 and EdU assay results confirmed that Ce-MBGNs (1 wt/v% extract) were non-cytotoxic and could significantly promote LECs proliferation.

In our previous study, Ce-MBGNs were proven to be non-cytotoxic against fibroblast cells at 1 mg/mL (0.1 wt/v%) [6], in which a direct cell culture test was performed. Here, we used an indirect test to evaluate the cytotoxicity of Ce-MBGNs against LECs as the target application of Ce-MBGNs is to act as a filler of nanocomposites. In this application, BGs mainly exert therapeutic activities on cells indirectly by releasing active ions. The cytotoxicity of Ce-MBGNs is thus mainly related to the released active ions (Si, Ca, and Ce) that are known for their concentration-dependent biological activities, including cytotoxicity [28,32]. We thus investigated the ionic concentrations of conditioned media (extract of 1 wt/v% MBGNs and Ce-MBGNs) by ICP-OES. The results showed that the conditioned media of MBGNs contained ~225 mg/L of Ca ions and ~70 mg/L of Si ions, while the conditioned media of Ce-MBGNs contained ~305 mg/L of Ca ions, ~61 mg/L of Si ions and ~113 μg/L of Ce ions. In the current study, more Ca ions than Si ions were detected in the conditioned media, which could be due to the burst release of Ca ions in 24 h and the presence of intrinsic Ca ions in DMEM [6,33]. A significantly lower concentration of detected Ce ions could be explained by the interactions between Ce ions and species (e.g., phosphate, sulfate groups) in culture media inducing (insoluble or low solubility) deposit formation [28,34]. Our results indicate that ionic concentrations of ~305 mg/L Ca ions, ~61 mg/L Si ions and ~113 μg/L Ce ions were non-cytotoxic against LECs, and they promoted cell proliferation. It is known that the cytotoxicity of BG extracts is concentration-dependent. However, the quantitative correlation between ionic concentration and LEC responses should be investigated in detail in future studies. Cerium oxide has been reported to effectively enhance the proliferation and migration of vascular endothelial cells [30]. Here, the presence of Ce in the extracts of Ce-MBGNs could be the main reason leading to the significantly enhanced LECs proliferation. However, the positive effects of Si and Ca ions on LECs should not be neglected, given the well-known effects of these ions on the proliferation and migration of vascular endothelial cells [35]. Nevertheless, the conditioned media (extract of 1 wt/v% MBGNs) only containing Si and Ca ions could not significantly enhance LEC proliferation in comparison to the control and VEGF-C group. However, the simultaneous presence of Si, Ca, and Ce ions in conditioned media promoted LEC viability and proliferation. By tailoring the release of these active ions (including the concentration of ions, release rate) from Ce-MBGNs, the biological behavior of LECs can be controlled toward promoted lymphangiogenesis.

### 3.3. Ce-MBGN Enhanced LECs Migration

**Figure 4:**
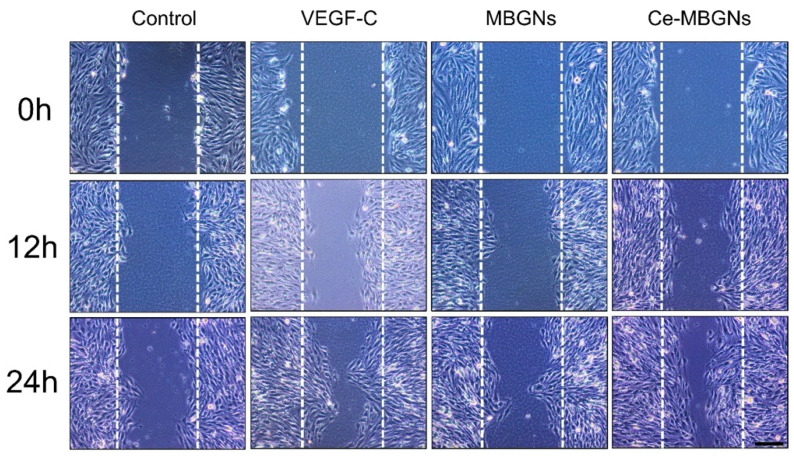
The scratch wound healing assay showing the migration ability of LECs cultured with different conditioned culture media, namely VEGF-C (50 ng/mL), MBGNs (1 wt/v% extract), and Ce-MBGNs (1 wt/v% extract). Scale bar = 200 μm. The experiment was performed at least in triplicate independently. The white dotted lines indicate the area of initial injury (scratch).

**Figure 5:**
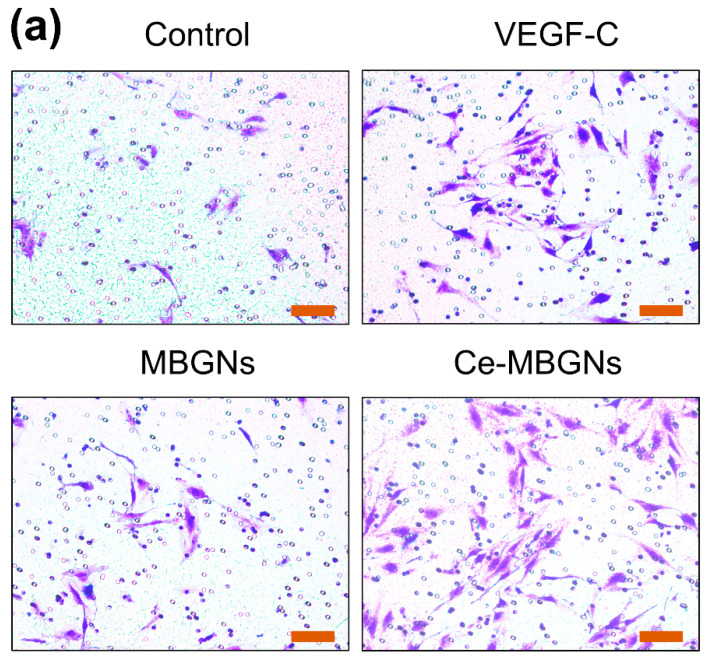

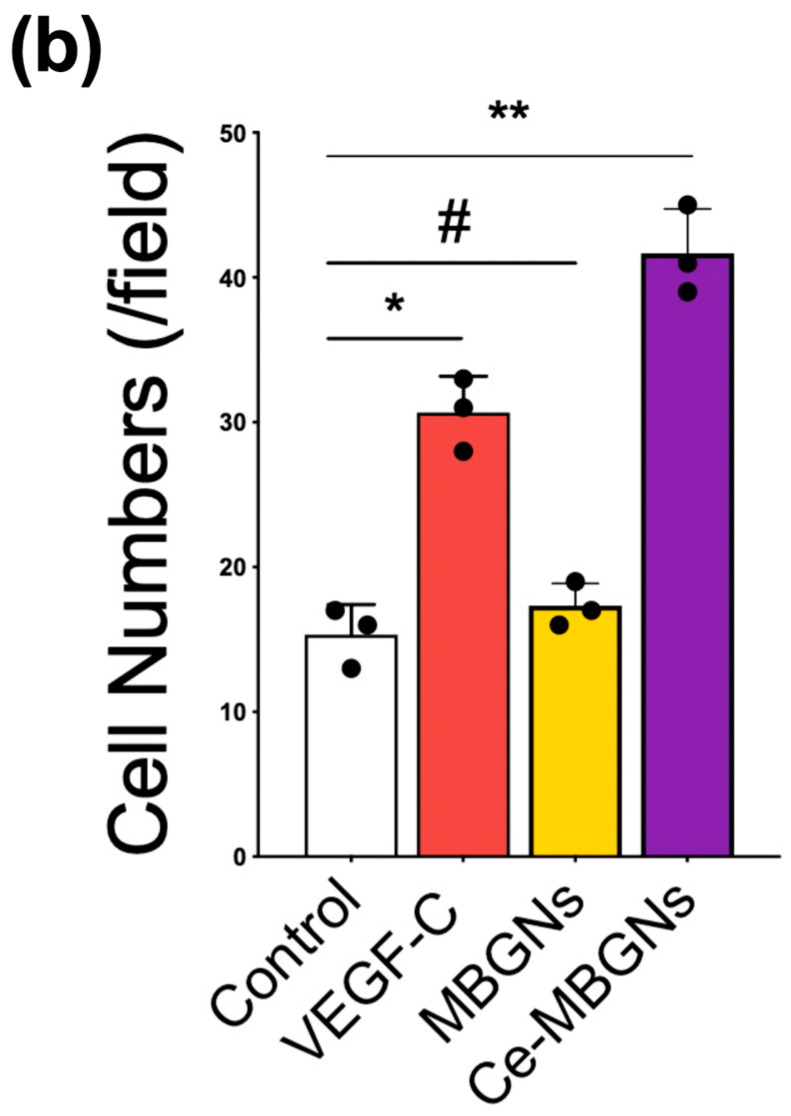
Images of Transwell migration assays (**a**) and quantitative analysis of migrated cell numbers (**b**) showing the migration ability of LECs cultured with conditioned media VEGF-C (50 ng/mL), MBGNs (1 wt/v% extract), and Ce-MBGNs (1 wt/v% extract). Scale bar = 200 μm. The experiment was performed at least in triplicate independently with three technical repeats. *p* < 0.05 (*), *p* < 0.01 (**) and *p* > 0.05 (#).

The in vitro scratch wound healing assay was performed to evaluate the effects of conditioned media on LEC migration, a key process of lymphangiogenesis. We investigated the ability of LECs to migrate into the wounded area over time (Figure 4). At 12 h after the scratch, LECs in the VEGF-C and Ce-MBGN groups started to display increased dynamic migration compared to that in the MBGN and control groups. After 24 h of culture, the wounded area in the VEGF-C and Ce-MBGNs groups was reduced compared to the area in the control group, indicating the positive effects of VEGF-C and Ce-MBGNs on LECs migration. However, MBGNs did not enhance the migration of LECs compared to the control. Ce-MBGNs enhanced migration of LECs similarly to the VEGF-C group after 12 h of culture, while they enhanced cell migration to a greater extent after 24 h compared to the VEGF-C group. Moreover, we performed a Transwell migration assay to investigate the effects of Ce-MBGNs on cell migration. As seen in Figure 5, no significant difference in the number of migrated cells between the MBGNs group and control could be observed. In comparison to the control, the VEGF-C and Ce-MBGNs groups significantly enhanced the number of migrated LECs. Moreover, Ce-MBGNs could stimulate more LECs migration than VEGF-C, which is consistent with the results of the scratch wound assay (Figure 4). The results of the scratch wound and Transwell migration assays proved the capacity of Ce-MBGNs to promote the migration of LECs.

BGs have been widely reported for their stimulating effects on tissue regeneration target cells, including osteoblasts, fibroblasts, and vascular endothelial cells [36,37]. In the present study, we evidenced for the first time that Ce-containing MBGNs could enhance the migration of LECs. VEGF-C has been known to enhance LECs migration probably due to the central role of the VEGF-C/VEGFR-3 signaling pathway in LEC behavior [38]. Cerium oxide has been reported to enhance the migration of vascular endothelial cells [30]. Therefore, the presence of Ce ions in the Ce-MBGNs group was thought to be the main factor contributing to the enhanced cell migration. However, the cellular pathway behind this phenomenon should be investigated in more detail.

### 3.4. Ce-MBGNs Improved Lymphatic Vascular Network Formation

**Figure 6:**
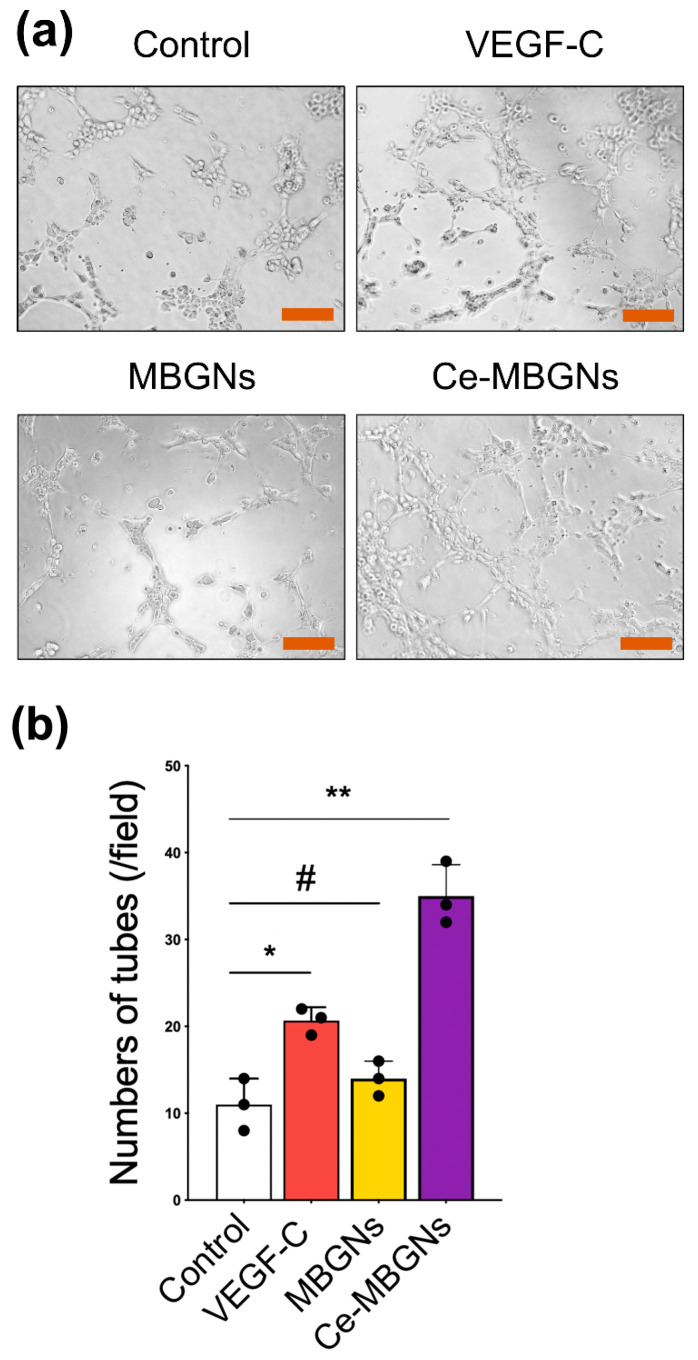
Tube-formation assessed using the Matrigel assay. The results were recorded after 3 h. (**a**) Tube formation of LECs cultured with conditioned media VEGF-C (50 ng/mL), MBGNs (1 wt/v% extract), and Ce-MBGNs (1 wt/v% extract) and the control. Scale bar = 100 μm. (**b**) LEC tube formation was quantified using the images. The experiment was performed at least in triplicate independently with three technical repeats. *p* < 0.05 (*), *p* < 0.01 (**) and *p* > 0.05 (#).

We further investigated the influence of Ce-MBGNs on lymphatic vascular network formation. The network formation capacity of LECs was assessed by determining the number of formed tubes. As shown in Figure 6, the tube formation of LECs was substantially improved in the presence of VEGF-C compared to the control group. No significant difference between the MBGNs group and the control was observed, suggesting the limited effects of MBGNs (1 wt/v% extract) on lymphatic vascular network formation. In contrast, the Ce-MBGNs group led to remarkably enhanced tube formation in comparison to the control and MBGNs group. Moreover, the quantitative analysis of the number of formed tubes (Figure 6b) indicated that Ce-MBGNs could improve lymphatic vascular network formation to a greater extent than VEGF-C (50 ng/mL), which is a well-known growth factor able to stimulate lymphatic vascular network formation [15].

### 3.5. Ce-MBGNs Regulated Lymphangiogenesis through the HIF-1α/VEGFR-3 Pathway

**Figure 7:**
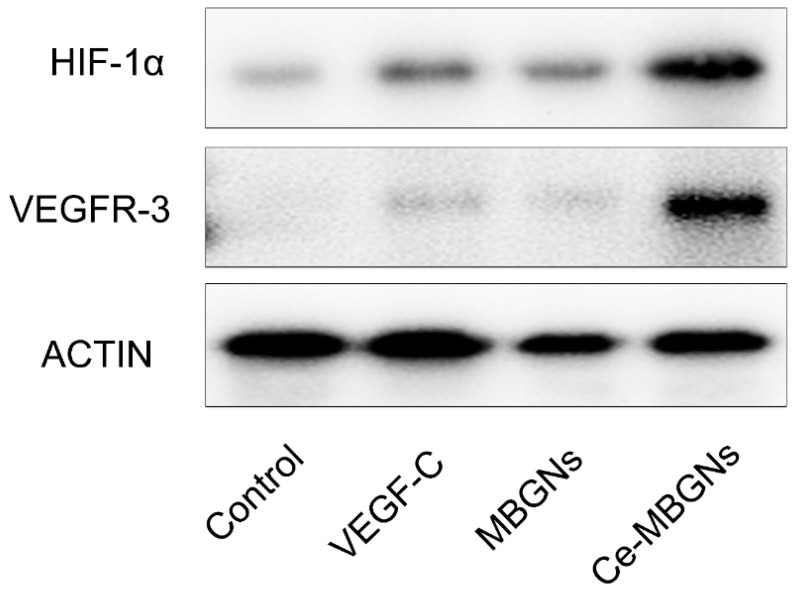
Western Blot results of LECs stimulated by VEGF-C (50 ng/mL), MBGN (extract of 1 wt/v%) and Ce-MBGN (extract of 1 wt/v%) for core HIF-1α and VEGFR-3 markers.

Expression of VEGFR-3 can be found in all endothelia during development, but later, VEGFR-3 becomes mainly found in lymphatic cells [15]. VEGFR-3 is thought to play a significant role in the proliferation and migration of LECs [39]. The VEGF-C/VEGFR3 signaling pathway is a dominant regulator of lymphangiogenesis [40]. Hypoxia-inducing factor-1α (HIF-1α) can be activated to promote lymphatic metastasis by regulating VEGF-C [41]. Cerium-containing biomaterials, such as cerium oxide and cerium-doped bioceramics, have been shown to enhance angiogenesis through modulating the intracellular oxygen environment and inducing the hypoxia condition locally [42,43]. Cerium-containing biomaterials can thus stabilize HIF-1α and increase VEGF secretion. Based on these results published in the literature, we hypothesized that Ce-MBGNs could regulate lymphangiogenesis by influencing HIF-1α and VEGFR-3. We used Western Blot to investigate the expression of HIF-1α and VEGFR-3 in LECs cultured with the conditioned media (50 ng/mL VEGF-C, extract of 1 wt/v% MBGNs, extract of 1 wt/v% Ce-MBGNs). As shown in Figure 7, the expression of HIF-1α and VEGFR-3 in the VEGF-C group was significantly increased compared to the control group. MBGNs were shown to slightly enhance the expression of HIF-1α in comparison to the control, probably because of the effects of Si and Ca ions. Previous studies have shown that the release of Si and Ca ions from BGs could stimulate the expression of HIF-1α from gene to protein levels [44,45]. Importantly, we observed a significant elevation of HIF-1α and VEGFR-3 expression in the Ce-MBGN group compared to the control. These results suggest that Ce-MBGNs can induce lymphangiogenesis probably through the HIF-1α/VEGFR-3 pathway. Ce-containing materials (representatively cerium oxide nanoparticles) have been well-known for their redox activity that is induced due to the quick alternation between Ce^4+^ and Ce^3+^ oxidation states. Ce-based compounds can thus act as multienzyme mimic or scavengers of reactive oxygen species (ROS) [43]. Incorporation of Ce can also endow BGs with such ROS-scavenging properties [7]. It has been reported that Ce could stabilize HIF-1α in endothelial cells by modulating oxygen levels in the intracellular environment and consequently induce angiogenesis [42]. Our results indicate that the enhanced lymphangiogenesis is probably linked to the capability of Ce in stabilizing HIF-1α in LECs, not directly related to the ROS-scavenging properties of Ce. However, Ce-MBGNs are also expected to be able to promote lymphangiogenesis by scavenging ROS in the condition of ROS overexpression.

In the present work, we showed that Ce-MBGNs can effectively promote lymphangiogenesis, as evidenced by the enhanced viability, proliferation, migration of LECs and lymphatic vascular network formation. Lymphangiogenesis has been a therapeutic target for chronic wound treatment [14,46]. Current strategies mainly focus on the delivery of growth factors (e.g., VEGF-C) using biomaterials (e.g., hydrogels) to stimulate lymphangiogenesis [14,15,19,24]. However, several limitations are associated with the strategy of delivering growth factors, for example, the short half-life and instability of growth factors. Our results for the first time showed the potential of BGs (specifically Ce-MBGNs) intrinsically stimulating lymphangiogenesis by releasing biologically active ions. Compared to the results of the MBGNs group, it could be concluded that Ce played an important role in lymphangiogenesis. However, the promoted lymphangiogenesis resulted from the synergistic effects of Si, Ca, and Ce ions released by Ce-MBGNs. The effect of a specific ion on in vitro lymphangiogenesis is still not clear. Further studies focusing on the mechanism of a single ion on lymphangiogenesis should be performed. Nevertheless, the use of Ce-MBGNs to stimulate lymphangiogenesis is a particularly appealing strategy for promoting lymphatic regeneration, as this approach avoids using unstable and expensive growth factors. In addition, antioxidant, and anti-inflammatory properties of Ce-MBGNs are also beneficial for the healing of chronic wounds under inflammation conditions [6]. Our study provides an alternative strategy for promoting lymphangiogenesis using BGs and expands the therapeutic applications of BGs in lymphangiogenesis-related tissue regeneration (including wound healing) and diseases.

## 4. Conclusions

In the present study, we investigated for the first time the effects of binary SiO_2_-CaO MBGNs and Ce-containing MBGNs (Ce-MBGNs) on lymphangiogenesis in vitro. Our results revealed that the extracts of Ce-MBGNs (1, 5, or 10 wt/v% in DMEM) were non-cytotoxic against LECs. Conversely, the extracts could promote the proliferation of LECs. In addition, the extracts of Ce-MBGNs (1 wt/v%) could enhance the migration of LECs and promote lymphatic vascular network formation compared to the blank control and the VEGF-C (50 ng/mL) group. However, the extracts of Ce-free MBGNs (1 wt/v%) did not promote the biological responses of LECs. The Western blot results suggested that lymphangiogenesis was probably induced through the HIF-1α/VEGFR-3 pathway due to the oxygen modulation capability of Ce-MBGNs. Overall, our results indicate that Ce-MBGNs can enhance lymphangiogenesis in vitro. Our study also provides a potential strategy to develop advanced BG-based medical devices for wound healing through stimulating lymphangiogenesis. However, understanding the specific roles of each ion (Si, Ca, Ce) in the lymphangiogenesis process and the concentration-dependent effects of ions still require further investigation in future works.

## Data Availability

The data used to support the findings of this study are available from the corresponding author on request.
